# Pseudo-subarachnoid haemorrhage due to chronic hypoxaemia: case report and review of the literature

**DOI:** 10.1186/s12883-014-0219-7

**Published:** 2014-11-18

**Authors:** Maximilian Patzig, Christoph Laub, Hendrik Janssen, Lorenz Ertl, Gunther Fesl

**Affiliations:** Department of Neuroradiology, University of Munich - Grosshadern Hospital, Marchioninistr. 15, 81377 Munich, Germany; Department of Neurology, University of Munich - Grosshadern Hospital, Marchioninistr. 15, 81377 Munich, Germany

**Keywords:** Subarachnoid haemorrhage, Hypoxaemia, Hypoxia, Computed tomography, Magnetic resonance imaging

## Abstract

**Background:**

The specificity of computed tomography (CT) for subarachnoid haemorrhage (SAH) is very high. However, physicians should be aware of rare false positive findings, also referred to as “pseudo-SAH”. We present an unusual case in which such a finding was caused by chronic hypoxaemia.

**Case presentation:**

A 37-year-old male patient presented with headaches. His CT-scan showed multiple confluent subarachnoid hyperattenuations, which mimicked SAH. However, the headache was chronic and had no features typical for SAH. The patient suffered from severe chronic hypoxaemia due to congenital heart failure. On CT-angiography diffuse intracranial vessel proliferation was found and laboratory results revealed a highly raised level of haematocrit, which had both probably developed as compensatory mechanisms. A combination of these findings explained the subarachnoid hyperdensities. Magnetic resonance imaging (MRI) showed no signs of SAH and visualized hypoxaemia in cerebral veins. A diagnosis of pseudo-SAH was made. The patient’s symptoms were likely due to a secondary headache attributed to hypoxia and/or hypercapnia. Therapy was symptomatic.

**Conclusions:**

Severe chronic hypoxaemia should be recognised as a rare cause of pseudo-SAH. Clinical evaluation and MRI help differentiate SAH from pseudo-SAH.

## Background

In the diagnosis of subarachnoid haemorrhage (SAH), non-enhanced computed tomography (NECT) is known to have a high sensitivity and an even higher specificity, which has recently even been reported to be 100% [1]. Thus, false positive CT findings, sometimes called pseudo-SAH, are rare. We present an unusual case of pseudo-SAH which had its origin in chronic hypoxaemia.

## Case presentation

A 37-year-old patient had undergone NECT in a radiological practice because of headaches. The scan showed bilateral multiple hyperattenuations in the hemispheral sulci and basal cisterns. A diagnosis of SAH was made and the patient was referred to our emergency department.

On admission, the patient reported that he had been suffering from progressively worsening headaches for about six months. The pain was of a dull, pressing quality and localized occipitally, radiating to parietal regions bilaterally. The headache was most severe in the morning hours, with a maximum of 7 on a pain scale of 0–10.

Medical history revealed postnatal major cardiac surgery due to a transposition of the great arteries. Moreover, several pulmonary embolisms were known and the patient was on phenprocoumon as a long-term therapy.

**Figure 1:**
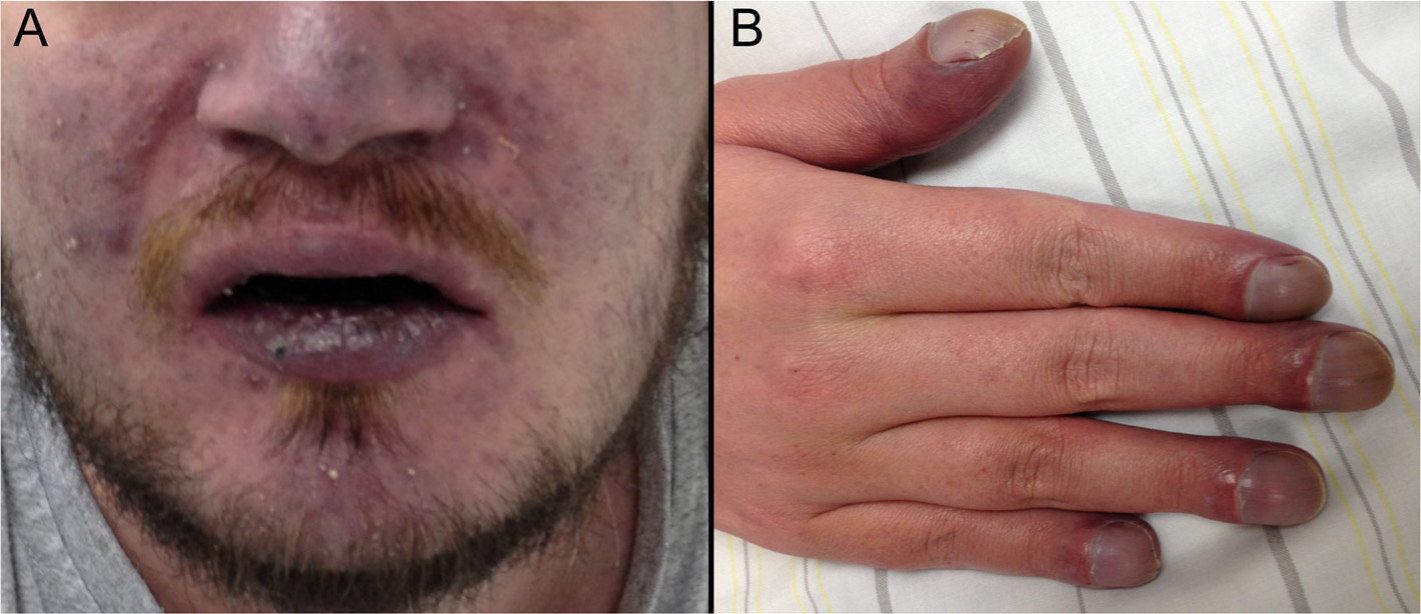
**Clinical signs of chronic hypoxaemia. A**: Facial cyanosis. **B**: Clubbed fingers and hippocratic nails.

On clinical examination, marked cyanosis, clubbed fingers and hippocratic nails were found, indicating chronic hypoxaemia (Figure 1). The clinical neurological examination was negative. Oxygen saturation measured by pulse oximetry was 70-74%, which the patient said were his “normal” values. Laboratory results notably showed a haematocrit level of 74.8% and a haemoglobin level of 22.1 mg/dl. International Normalized Ratio (INR) was 8.2 and Partial Thromboplastin Time (PTT) was 114 s.

**Figure 2:**
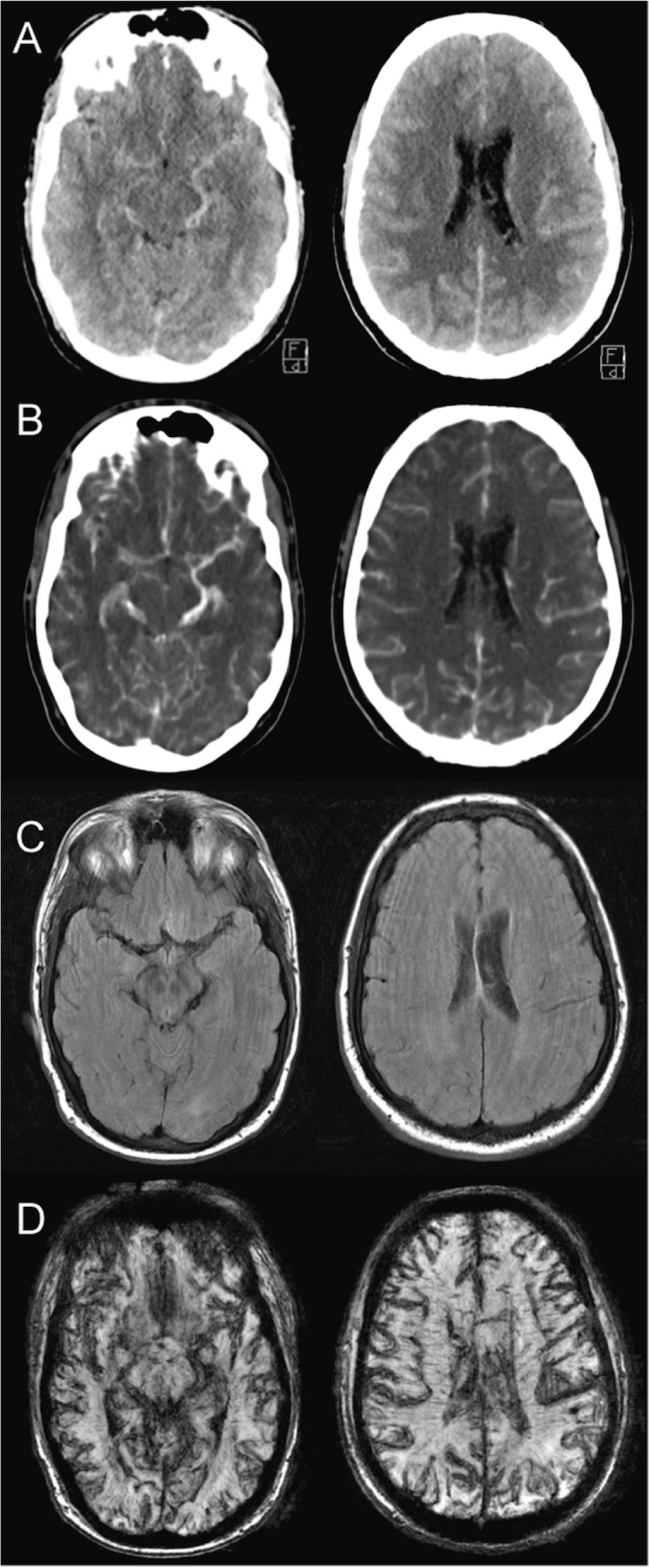
**Imaging.** Exemplary images of matching slice plains. All images represent 5 mm slices, with images in A, B and D being reconstructions of thinner source images. MR images are of reduced quality due to motion artefacts. **A**: Non-enhanced CT: Diffuse subarachnoid hyperattenuations mimicking SAH. **B**: CT-angiography: Sulcal and basal veins dilated and increased in number. **C**: MRI, FLAIR sequence: No findings of SAH. **D**: MRI, SWAN sequence: Markedly hypointense depiction of cerebral and subarachnoidal veins due to hypoxaemia.

CT imaging was performed, with the repeated NECT showing the same findings as the scan performed at the external radiology practice (Figure 2A). CT-angiography (CTA) of the intracranial vessels showed no aneurysm or vascular malformation, but dilated veins ubiquitous in the subarachnoid space (Figure 2B). The hyperattenuations on NECT correlated with the course of the vessels found on CTA. Thoracic CT-angiography revealed atrial and ventricular septum defects and a pulmonary trunk fed by both ventricles. The pulmonary arteries were severely dilated, while the aorta, springing regularly from the left ventricle, had a small diameter. The right atrium was enlarged and the right ventricle was hypertrophic. Severe chronic pulmonary thromboembolism was shown.

As the diagnosis of SAH was now strongly doubted, a 3-Tesla MRI scan (Signa HDx scanner, GE, Milwaukee, WI, USA) was performed for further clarification. The protocol included FLAIR, PD and T2*-weighted sequences as well as susceptibility-weighted angiography (SWAN) (Figure 2C and D). SWAN revealed the intracranial veins to be very hypointense and with a large diameter, due to the susceptibility effect caused by the increased fraction of deoxyhaemoglobin in the venous blood of the patient (Figure 2D). The image quality was markedly reduced because of motion artefacts. It was sufficient, however, to rule out the diffuse CT-hyperattenuations representing subarachnoid blood. There were no findings indicative of acute SAH or superficial haemosiderosis.

In view of these findings, the diagnosis of diffuse SAH was rejected. We therefore refrained from a lumbar puncture (particularly in view of the impaired coagulation) or digital subtraction angiography (DSA). The patient obviously had chronic hypoxaemia due to the complex cardiopulmonary situation with major intermixture of the blood of the systemic and pulmonary circulations. Compensatory polycythaemia had developed. Generalized proliferation and dilatation of intracranial veins likely were another compensatory mechanism to maintain sufficient cerebral oxygen supply. The increased haematocrit in combination with the prominent vessels can explain the subarachnoid hyperdensities, which mimicked severe SAH. The headache had no features characteristic of SAH. It was classified as chronic tension headache or headache in relation to hypoxia and/or hypercapnia. A symptomatic therapy was recommended.

## Conclusions

Hyperattenuations of the sulci and/or basal cisterns on NECT in most cases represent SAH. In some patients, however, the underlying pathology is different. These cases are also referred to as “pseudo-SAH”. This phenomenon has been recognized in the literature in several case reports and rather small case series. In rare cases, leptomeningeal disesases such as purulent meningitis [2,3], meningeal leukaemia [4] and gliomatosis [5] have been reported to mimic SAH. Contrast agent neurotoxicity is another rare cause of CT hyperdensities which can be mistaken for SAH. Obviously, however, this can only be a differential diagnosis if contrast has been applied, usually via DSA [6-8]. Moreover, pseudo-SAH has been encountered in patients with intracranial hypotension [9-11], cerebellar infarctions [12,13] and bilateral subdural haematoma [14,15]. Most reports of pseudo-SAH, however, exist on patients with severe generalized cerebral edema. These include rare cases of toxic encephalopathy after valproic acid [16] or bee sting poisoning [17], but mostly patients with severe hypoxic or anoxic encephalopathy [18-25]. Avrahami et al. [25] performed lumbar punctures in 26 comatose patients with generalized cerebral edema and CT diagnosis of SAH, and found no bloody or xanthochromic cerebrospinal fluid in any of them. Yuzawa et al. [18] report pseudo-SAH in eight of 45 consecutive patients who underwent CT scans after resuscitation from cardiopulmonary arrest. Pseudo-SAH was associated with severe cerebral edema in all of these patients. Explanations for the paradoxical imaging findings include a narrowing of the sulci and cisterns and a shift of cortex into the subarachnoid spaces, along with pathologically reduced density of adjacent brain parenchyma and vessel congestion.

In our case of pseudo-SAH, we believe the origin of the hyperattenuations in the subarachnoid space to be different from the reports mentioned above, which mostly describe acute encephalopathies. Our patient suffered from chronic hypoxaemia, and there were no signs of cerebral edema on the CT and MRI scans. So there was no thinning of the sulci and no loss of density of the cortex which could explain the sulcal hyperdensities. We believe that one reason for the hyperattenuations in the CT scan of our patient was his extremely elevated haematocrit level. The density of intravascular blood correlates linearly positively with the haematocrit level [26-30]. Increased vessel density due to polycythaemia is known to be a pitfall in the diagnosis of hyperdense middle cerebral artery signs [31,32] or venous sinus thrombosis [33,34]. Brief reports also exist on diffuse vessel hyperdensities caused by polycythaemia [26,35]. The other major factor in our case were proliferated and dilated sulcal and basal veins, which likely had developed to compensate for the chronic hypoxaemia. So we hypothesize that numerous and prominent vessels which appeared hyperdense because of a highly raised haematocrit level had caused the SAH-like picture.

The use of Hounsfield unit (HU) measurements to discriminate between pseudo-SAH and SAH in patients with post-resuscitation encephalopathy has been discussed, with lower HU values presumed to indicate pseudo-SAH [18]. Our patient had relatively high HU values of 52 – 62 (measured in the sylvian fissure). However, given the different aetiology of pseudo-SAH in our case and the haematocrit level of 75%, we did not interpret these measurements as favouring a diagnosis of SAH.

The case illustrates a very rare differential diagnosis of SAH on CT scans. It should be considered particularly if the clinical symptoms are not typical for SAH and the patient has severe polycythaemia. When such a case is suspected, we suggest imaging like CT-angiography and particularly MRI to confirm the diagnosis. In low-risk patients, a lumbar puncture to definitely exclude SAH should be discussed. In our severely coagulopathic patient, however, we wanted to avoid invasive diagnostics. The MRI scan had shown that the diffuse hyperdensities found on NECT did not represent blood. This imaging finding along with the clinical picture, which was not indicative of SAH, gave us enough diagnostic confidence to refrain from invasive methods.

The case also underlines the ability of the SWAN MRI sequence to visualize a reduced oxygen content in cerebral veins. The veins appeared markedly hypointense in a patient with obvious severe chronic hypoxaemia. Susceptibility-weighted imaging (SWI) is known to be sensitive to different oxygenation statuses [36,37], and we found that SWAN, a T2*-weighted multiple-echo gradient-echo sequence which differs from SWI by using only magnitude information, also has that quality. Further studies are needed to explore clinical applications of this potential.

The headache of our patient fulfilled the criteria of chronic tension headache. However, considering the underlying disorders of the patient and the associations of headaches with polycythaemia, chronic lung diseases and hypoxia as experienced at great altitude [38-42], a secondary headache attributed to hypoxia/hypercapnia with the phenotype of tension headache was the more likely diagnosis. Either way, therapy could only be symptomatic.

### Consent

Written informed consent was obtained from the patient for publication of this case report and any accompanying images. A copy of the written consent is available for review by the Editor of this journal.
